# Single-Crystal NMR Spectroscopy of Spin *I* = 3: ^10^B-NMR of Hambergite, Be_2_BO_3_OH

**DOI:** 10.3390/molecules31142554

**Published:** 2026-07-22

**Authors:** Jennifer Steinadler, Christian Minke, Thomas Bräuniger

**Affiliations:** Department of Chemistry, University of Munich (LMU), Butenandtstr. 5-13, 81377 Munich, Germany; jennifer.steinadler@cup.uni-muenchen.de (J.S.); christian.minke@cup.uni-muenchen.de (C.M.)

**Keywords:** ^10^B-NMR, NMR interaction tensors, single crystal

## Abstract

A monocrystal of the natural mineral hambergite, Be_2_BO_3_OH, is studied by ^10^B-NMR spectroscopy. The nuclide ^10^B possesses spin I=3, and thus for a single boron site in a periodic solid, the ^10^B spectrum is composed of three doublets. For the four inversion-related pairs of boron sites existing in the crystal structure of hambergite, orientation-dependent ^10^B spectra are recorded and both the chemical shift and electrical field gradient (EFG) tensor are extracted. The resulting numerical values are in very good agreement with a previous ^11^B-NMR study of the same sample. Comparing the line widths of the NMR resonances of ^11^B against ^10^B, the concept of inherently better resolution being available from ^10^B due to the scaling down of dipolar interactions is confirmed.

## 1. Introduction

The first solid-state NMR measurements of the nuclide ^10^B with spin I=3 were reported by Bray and co-workers in the 1970s [1,2,3,4]. However, the preferred isotope for NMR of boron is ^11^B with spin I=3/2, which possesses much higher natural abundance (80.1%, with the remaining 19.9% being ^10^B) and a resonance frequency almost triple that of the ^10^B isotope. For this reason, the use of ^11^B-NMR as an analytical tool for solids, also involving P.J. Bray, was pioneered earlier [5,6,7]. Although the spin properties of ^10^B look unfavourable at first sight, an argument can be made that quadrupolar interaction parameters can be extracted from spectra of this isotope to higher precision than from ^11^B [1]. First, dipolar broadening present in many systems adversely affects the line shapes, which are reduced because of the smaller gyromagnetic ratio of ^10^B. Second, the inner transitions of spin I=3 (m=0↔±1) span a much wider spectral range than the central transition of spin I=3/2 (m=+1/2↔−1/2). Most ^11^B-NMR studies have restricted themselves to evaluating this transition [8], as its intensity is much stronger than those of the satellite transitions. This advantage in resolution and precision becomes especially significant when investigating systems with a distribution of NMR interaction parameters, such as glasses; in fact, the interest in early ^10^B-NMR was mostly motivated by structural studies of borate glasses, and this seems to remain the main motivation in more recent publications [9,10,11], with some more exotic applications also reported [12,13]. In all the work on ^10^B published so far, theoretical descriptions and fitting algorithms have been focused on what are in effect polycrystalline samples, which deliver spectra with very broad lines. Invariably, these broad lines lead to loss of spectral resolution, and while this can be partly remedied with advanced methods such as overtone spectroscopy [14], improved resolution may always be obtained from monocrystalline samples [15,16], with the restriction that single crystals of sufficient size must be available.

In this work, the natural mineral hambergite is used as a model system to conduct a ^10^B-NMR study of a single crystal. The comparatively rare mineral hambergite, Be_2_BO_3_OH, appears in beryllium-bearing granite pegmatites as an accessory phase, and forms monocrystals of sufficient size. Single crystals of hambergite have been studied before extensively by ^1^H-NMR in order to analyse the properties of the one-dimensional proton chains in the crystal structure [17,18,19]. Characterisation of Be_2_BO_3_OH by single-crystal ^9^Be- and ^11^B-NMR, on the other hand, has been performed only very recently by our group [20]. The application of ^10^B-NMR to hambergite presented here was carried out on the same crystal specimen with identical orientation of the crystal on the goniometer axis (within experimental error; see below for details). This enables direct comparison between ^10^B- and ^11^B-NMR applied to boron atoms in identical crystallographic environment, allowing us to again test and verify the claims regarding superior accuracy of ^10^B-NMR [1] while taking advantage of the superior resolution and defined orientation dependence of single-crystal spectra. Before discussing these results, we briefly review the principles of NMR spectroscopy of spin I=3 in the solid state.

## 2. NMR Spectroscopy of Spin I=3 in the Solid State

Following the notation used in [16], the resonance frequency of a nuclear spin in a solid-state NMR experiment at high magnetic field may be expressed as a sum of the various interactions contributing to the energy levels of a spin system, with $\nu_{0}$ being the Larmor frequency and $\nu_{\mathrm{CS}}$, $\nu_{\mathrm{DD}}$, and $\nu_{J}$ being the contributions of the chemical shift, the direct dipolar and the indirect coupling, respectively, at some general orientation Ω:

(1) $$\nu \left(\Omega \right)=\nu_{0}+\nu_{\mathrm{CS}}\left(\Omega \right)+\nu_{\chi }\left(\Omega \right)+\nu_{\mathrm{DD}}\left(\Omega \right)\;\left[+\nu_{J}\left(\Omega \right)\right]$$

Regarding the dipolar interaction described by $\nu_{\mathrm{DD}}$, it splits the respective resonances of an isolated spin-1/2 pair *I* and *S* into a doublet with frequencies $\nu^{+}$ and $\nu^{-}$. These are placed symmetrically around the unaffected resonance position, with the magnitude of the splitting scaled by the distance $r_{IS}$ between the two spins and with β being the angle between the vector connecting the two spins and the external magnetic field lines:

(2) $$\nu_{\mathrm{DD}}^{\pm }\left(\Omega \right)=\pm \frac{\mu_{0}\gamma_{I}\gamma_{S}\hbar }{8\pi^{2}r_{IS}^{3}}\cdot \frac{3\cos^{2}\beta -1}{2}$$

For nuclei with spin I>1/2, the dipolar interaction creates more complex multiplets, with each spin contributing 2I+1 energy levels. Also, in most samples the spins do not occur as isolated pairs but as part of a large coupled network, which for polycrystalline samples leads to severe broadening of the resonance lines. This is one aspect where ^10^B-NMR should have an advantage over ^11^B-NMR, as the gyromagnetic ratio of the former is only about one-third that of that of the latter [21]:

(3) $$\frac{\gamma {(}^{10}B)}{\gamma {(}^{11}B)}=\frac{2.8747\cdot 10^{7}\;\mathrm{rad}\;s^{-1}\;T^{-1}}{8.5847\cdot 10^{7}\;\mathrm{rad}\;s^{-1}\;T^{-1}}=0.3349$$

Consequently, the dipolar couplings experienced by ^10^B and the concomitant line broadening are scaled down by the same factor.

Another important interaction for ^10^B-NMR of solids is the quadrupolar interaction, described by the frequency $\nu_{\chi }$, which is usually treated as a perturbation to the main Zeeman interaction energy [22]:

(4) $$\nu_{\chi }\left(\Omega \right)=\nu_{\chi }^{\left(1\right)}+\nu_{\chi }^{\left(2\right)}+\nu_{\chi }^{\left(3\right)}+\ldots$$

The product of the nuclear property eQ (with *Q* being the quadrupole moment of the nucleus, $Q{(}^{10}B)=84.59$ mb [23]) and the largest eigenvalue $V_{33}$ of the electrical field gradient (EFG) tensor V is called the quadrupolar coupling constant χ:

(5) $$\chi =C_{Q}=\frac{eQ}{h}V_{33}=\frac{eQeq}{h}$$

The ‘quadrupolar frequency’ $\nu_{Q}$ [22] is another useful measure of the interaction strength; in contrast to χ, it scales with the magnitude *I* of the observed spin:

(6) $$\nu_{Q}=\frac{3e^{2}qQ}{2I(2I-1)h}=\frac{3\chi }{2I(2I-1)}$$

For ^10^B with I=3, this results in

(7) $$\nu_{Q}{(}^{10}B)=\frac{3\chi }{6(6-1)}=\frac{\chi }{10}$$

We also find it useful to assign a parameter *k* to each transition in the spin system, such that [16]:

(8) $$k=m\pm \frac{1}{2}\;\mathrm{for}\;|m\rangle \to |m\pm 1\rangle$$

With the above definitions, we can now proceed to specify the individual frequencies $\nu_{\chi }^{\left(i\right)}$ in Equation (4). With the general orientation Ω defined by the azimuthal angle α and polar angle β of the magnetic field vector in the principal axes system of the quadrupole coupling tensor Q (see below), the first-order contribution of transition *k* for a static sample is given by

(9) $$\nu_{\chi }^{\left(1\right)}\left(k\right)=k\frac{\nu_{Q}}{2}\left(3\cos^{2}\beta -1+\eta_{Q}\cos2\alpha \sin^{2}\beta \right),$$

where $\eta_{Q}$ is the quadrupolar asymmetry parameter calculated from the components of the diagonalised Q-tensor, which are ordered according to $|Q_{33}\left|\;\geq \;\right|Q_{22}\left|\;\geq \;\right|Q_{11}|$:

(10) $$\eta_{Q}=\frac{Q_{11}-Q_{22}}{Q_{33}}$$

In contrast to NMR spectra of nuclei with half-integer spins, where a central transition with k=0 exists, all transitions of spin I=3 are affected by the first-order contribution. The maximal resonance frequencies are observed for β=0, such that

(11) $$\nu_{\chi }^{\left(1,\max\right)}\left(k\right)=k\cdot \nu_{Q}=k\cdot \frac{\chi }{10}$$

The properties of the ^10^B resonances in a single-crystal spectrum at maximal displacement are listed in Table 1.

**Figure 1:**
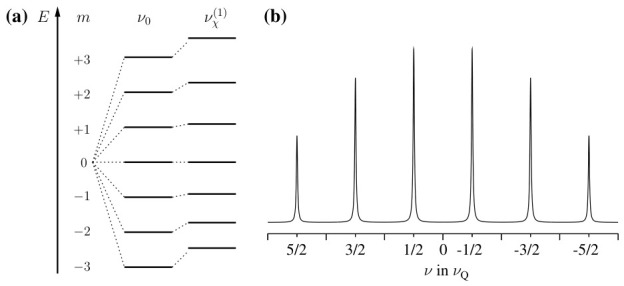
NMR spectroscopy of ^10^B with spin I=3: (**a**) energy levels for Zeeman and first-order quadrupole interaction; (**b**) schematic single-crystal spectrum for maximal displacement under first order, as given by Equation (11).

While the NMR spectrum of a nucleus with spin I=1 at a single crystallographic site in a monocrystal consists of a doublet, the corresponding spectrum of ^10^B with I=3 may be viewed as being composed of outer, middle, and inner doublets with relative intensities 3:5:6. If the quadrupolar interaction is sufficiently small to be fully described by first-order contribution only, then all resonances in the symmetric spectrum are spaced evenly, with the spacing for the maximal displacement being $\nu_{Q}=\chi /10$, as shown in the schematic spectrum in Figure 1b. When considering the quadrupole interaction to the first order only, the relevant terms of the resonance frequency in Equation (1) may also be expressed in a compact tensor notation (with $\vec{b}$ being the unit vector along the magnetic field lines and ${\vec{b}}^{t}$ its transpose), which for spin I=3 can be written as shown in Equation (12) below.

(12) $$\nu \left(\Omega \right)=\nu_{0}+\nu_{\mathrm{CS}}\left(\Omega \right)+\nu_{\chi }^{\left(1\right)}\left(\Omega \right)=\nu_{0}+{\vec{b}}^{t}\cdot \delta \cdot \vec{b}+\frac{k}{10}{\vec{b}}^{t}\cdot Q\cdot \vec{b}$$

In this notation, δ is the chemical shift tensor, the scaled trace of which is the isotropic chemical shift $\delta_{\mathrm{iso}}$. The quadrupole coupling tensor Q, on the other hand, is traceless, because it is directly related to the EFG tensor V by Q=(eQ/h)V:

(13) $$\begin{array}{ccc} \delta_{\mathrm{iso}}=\frac{1}{3}\sum_{i}\delta_{ii}\; & \; & \sum_{i}Q_{ii}=0 \end{array}$$

Both interaction tensors are symmetric, the chemical shift tensor δ by convention [24] and Q intrinsically because of the mixed partial derivatives in the definition of the EFG tensor [22]. In an arbitrary coordinate system designated as xyz, these two tensors have the following general form:

(14) $$\delta^{xyz}=\left(\begin{array}{ccc} \delta_{xx} & \delta_{xy} & \delta_{xz}\\ \delta_{xy} & \delta_{yy} & \delta_{yz}\\ \delta_{xz} & \delta_{yz} & \delta_{zz} \end{array}\right)\;Q^{xyz}=\left(\begin{array}{ccc} Q_{xx} & Q_{xy} & Q_{xz}\\ Q_{xy} & Q_{yy} & Q_{yz}\\ Q_{xz} & Q_{yz} & Q_{zz} \end{array}\right)$$

In single-crystal NMR experiments, the dependence of the resonance frequencies on the orientation Ω of the crystal relative to the magnetic field is systematically traced. This is usually done by mounting the crystal on a goniometer mechanics, rotating it stepwise by an angle $\varphi_{i}$, and recording a spectrum for each orientation. In our experimental setup, the goniometer axis is oriented perpendicular to the external magnetic field, which is the most common design, with other designs existing [15]. The rotation of the crystal (and the crystal-frame fixed interaction tensors) about the perpendicular axis makes the individual resonances follow harmonic functions of the following type [25]:

(15) $$\nu \left(\varphi_{i}\right)=A+B\cos2\varphi_{i}+C\sin2\varphi_{i}\;\left(+G\cos4\varphi_{i}+H\sin4\varphi_{i}\right)$$

The higher harmonics evolving with 4φ only show up when quadrupolar coupling effects are so strong that the first-order corrections $\nu_{\chi }^{\left(1\right)}$ do not suffice to describe the observed spectrum, and second-order contributions $\nu_{\chi }^{\left(2\right)}$ need to be included as well; see Equation (4). For ^10^B resonances of a static sample, these second-order contributions may be written as

(16) $$\nu_{\chi }^{\left(2\right)}\left(k^{2}\right)=-\frac{\chi^{2}}{600\nu_{0}}\left[\left(\frac{45}{4}-3k^{2}\right)g\left(\alpha ,\beta ,\eta_{Q}\right)-6k^{2}f\left(\alpha ,\beta ,\eta_{Q}\right)\right],$$

where the functions *g* and *f* have terms depending on $\cos^{4}\beta$ and $\cos^{2}\beta$, with the dependencies on α and $\eta_{Q}$ encapsulated in the coefficients $A^{\left(2\right)},B^{\left(2\right)},\ldots$, which are given in full in Appendix A.1.

(17) $$\begin{array}{c} \begin{array}{cc} g(\alpha ,\beta ,\eta_{Q}) & =A^{\left(2\right)}\cos^{4}\beta +B^{\left(2\right)}\cos^{2}\beta +C^{\left(2\right)}\\ f(\alpha ,\beta ,\eta_{Q}) & =D^{\left(2\right)}\cos^{4}\beta +E^{\left(2\right)}\cos^{2}\beta +F^{\left(2\right)} \end{array} \end{array}$$

Within the framework of equations given above, we can now proceed to analyse the ^10^B-NMR spectra of a single crystal of hambergite.

## 3. Results and Discussion

### 3.1. Boron NMR of Hambergite

**Figure 2:**
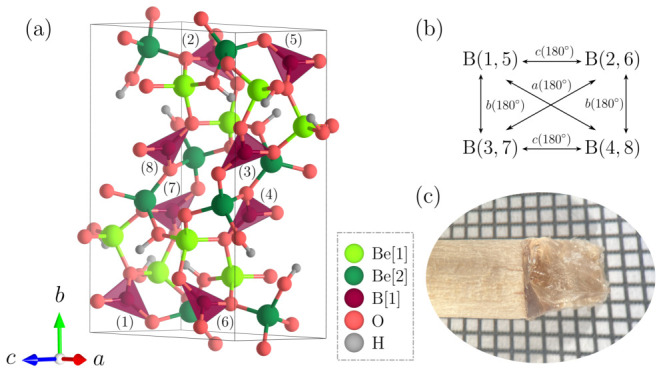
(**a**) Representation of the unit cell of hambergite, Be_2_BO_3_OH, in the standard setting Pbca, space group no. 61 (graphics generated with the Vesta 3 program [32]); the boron atoms, labelled (1) to (8), occupy Wyckoff position 8c and are connected by inversion centres and glide planes. (**b**) Summary of the NMR-relevant symmetry relations between the four inversion-connected pairs of boron atoms. (**c**) Single crystal of hambergite from Sahatany Valley/Madagascar, mounted on a wooden goniometer axis.

To understand boron NMR of hambergite, an important question is the number of expected resonances in a single-crystal spectrum, which of course is intrinsically linked to the crystal structure of Be_2_BO_3_OH. There have been several diffraction studies of hambergite over the years [26,27,28,29], including a recent one from our group [20]. All studies agree that the mineral crystallises in the orthorhombic and centrosymmetric space group no. 61, with lattice parameters and atomic coordinates always reported in the standard setting *Pbca*. Figure 2a shows a view of the unit cell of hambergite, where boron occupies a site with a Wyckoff multiplicity of eight. Since the boron atoms are pairwise related by inversion, four magnetically inequivalent pairs of ^10^B respectively ^11^B atoms exist, with their additional symmetry relations summarised in Figure 2b. These four pairs are observable in the corresponding NMR spectra; however, when attempting to relate these NMR signals to atoms in the unit cell, a number of problems arise. For orthorhombic systems such as hambergite, several choices for the setting of the unit cell exist, all being fully equivalent in terms of interpreting the NMR spectra. Also, the problem of assigning NMR resonances to individual atoms in the crystal structure is of fundamental nature [20,30,31], and can only be resolved by resorting to additional information derived from ab initio calculations of the electron density or other orientation-dependent data such as dipolar couplings. For single-crystal NMR of boron in the hambergite structure, these problems and their solutions have been discussed extensively for ^11^B-NMR in our previous publication [20], and the interested reader is referred to it. In the current work, we use these solutions and assignments for evaluating the ^10^B-NMR spectra without deriving them again.

### 3.2. Determination of the Quadrupole Coupling Tensor

**Figure 3:**
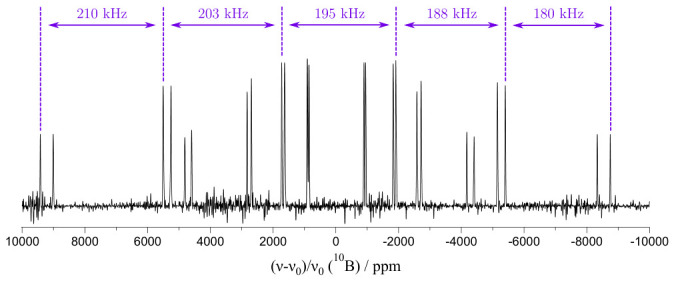
^10^B (with I=3) spectrum of a single crystal of hambergite, Be_2_BO_3_OH, at a nominal rotation angle of $\varphi =180^{\circ }$. Three separate spectra were acquired, with the transmitter set to approximately +6500, 0, and −6000 ppm, respectively, and the intensities scaled to match the theoretical predictions. Given above are the spacings between the resonances belonging to boron pair B(1,5), showing that the multiplet is not fully symmetric because of second-order effects as described by Equation (16).

A crystal specimen of hambergite was mounted on a wooden support axis, as shown in Figure 2c. This is the same crystal and for all practical purposes (see below) the same axis orientation as was used for ^11^B-NMR in the previous work [20]. Recording a complete ^10^B-NMR spectrum usually required the acquisition of several spectral windows, with a representative spectrum shown in Figure 3.

**Figure 4:**
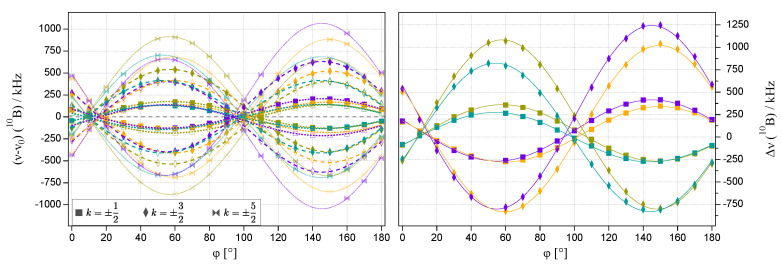
(**Left**): ^10^B rotation pattern of hambergite, recorded around a goniometer axis with the orientation $\theta_{g}={\left(38.98\pm 0.08\right)}^{\circ },\phi_{g}={\left(174.6\pm 0.1\right)}^{\circ }$ and an offset angle of $\varphi_{0}={\left(96.1\pm 0.1\right)}^{\circ }$ (see text for details). The lines represent harmonic functions of the type described by Equation (15), with the data belonging to the four magnetically inequivalent boron pairs (see Figure 2b) colour-coded in violet for B(1,5), teal for B(4,8), khaki for B(3,7), and orange for B(2,6). (**Right**): Splittings Δν according to Equation (18), with the same colour coding applied and transitions k=±5/2 excluded because of their low intensities; see text for details.

Subsequently, a set of spectra was acquired over the rotation angle range of $\varphi =0^{\circ }\ldots 180^{\circ }$, in steps of 10 degrees. Plotting the orientation-dependent resonance frequencies over φ gives the full ^10^B-NMR rotation pattern of hambergite, as displayed on the left of Figure 4.

The recorded ^10^B multiplets are not fully symmetric around zero, which is apparent from the multiplet spacings given on top of the spectrum shown in Figure 3. This asymmetry is caused by second-order contributions of the quadrupole interaction. The presence of these contributions makes it impossible to use Equation (12) to extract the quadrupole coupling tensor Q. However, they can be removed by taking the differences between the transition frequencies (Equation (1)) of the ±k doublets, resulting in the following expression:

(18) $$\Delta \nu \left(\varphi \right)=\nu \left(+k\right)-\nu \left(-k\right)=\Delta \nu_{\chi }^{\left(1\right)}\left(\pm k\right)=\frac{\Delta k}{10}{\vec{b}}^{t}\cdot Q\cdot \vec{b}$$

These differences Δν, which are also called splittings, are plotted on the right of Figure 4. In principle, for the various transitions *k* listed in Table 1, fit code expressions can be derived from Equation (18), which can be used to extract the quadrupole coupling tensor Q from the experimental data. However, in many spectra across the rotation pattern, the resonances belonging to the outer transitions with k=±5/2 were inadequately defined because of their comparatively low intensities, cf. Figure 1b. Therefore, these transitions were not included in the data fit. To first order, all transitions *k* are connected by constant factors; thus, only some redundancy is lost when excluding those with k=±5/2.

In order to relate the orientation of the magnetic field vector $\vec{b}$ to the rotation angle φ, the orientation of the goniometer axis $\vec{g}$ must be known. With sufficient data available from single-crystal NMR experiments, the orientation of $\vec{g}$ can be fitted from these data as well without resorting to additional crystal alignment techniques [16,33,34,35]. A brief overview of the equations needed for fitting the ^10^B-NMR data is given in Appendix A.2. As already mentioned above, for the current study we can take advantage of the previously published characterisation of the same crystal specimen by ^11^B-NMR [20] and make use of the goniometer axis orientation and tensor assignments derived there. Although the studied crystal remained glued to the goniometer axis rod, the rod itself had been removed from the experimental setup in between the ^11^B- and ^10^B-NMR measurements; thus, a small misalignment in both the direction of $\vec{g}$ and the zero-angle offset $\varphi_{0}$ was expected. In order to allow for correction of these small misadjustments, a ^11^B rotation pattern was additionally recorded while collecting the ^10^B data of Figure 4. Then, the splittings of this ^11^B rotation pattern (as shown in Appendix A.3) were fitted simultaneously with the ^10^B data, with only the goniometer axis direction and $\varphi_{0}$ as fit variables, i.e., with the ^11^B quadrupole coupling tensor elements fixed. The elements of the Q-tensor of ^10^B, on the other hand, were treated as free fit variables, resulting in the following tensor for the atom pair B(1,5).

(19) $$Q_{e}^{abc}/\;\mathrm{kHz}=\left(\begin{array}{ccc} 2310\pm 60 & 3972\pm 14 & 463\pm 12\\ . & 438\pm 12 & 288\pm 16\\ . & . & \left(-2748\pm 72\right) \end{array}\right)$$

Transforming the above tensor, which is expressed in the normalised orthorhombic crystal frame abc, into its own principal axes system (PAS) leads to a quadrupolar coupling constant of

(20) $$\chi {(}^{10}B)=\frac{e^{2}qQ{(}^{10}B)}{h}=Q_{33}^{\mathrm{PAS}}=5490\pm 40\;\mathrm{kHz}$$

Furthermore, with the elements of the quadrupole coupling tensor related to those of the electric field gradient tensor by $Q_{ij}=\left(eQ/h\right)V_{ij}$, the components of the EFG tensor can be calculated and compared to those derived from previous ^11^B-NMR measurements [20], with the results listed in Table 2.

From comparing the eigenvalues of the EFG tensors, it can be seen that the errors on the EFG tensor components are much larger for ^10^B than for ^11^B. This is chiefly due to the quality of the individual spectra, with ^10^B usually having a much worse signal-to-noise ratio (s/n). The usual way to improve the s/n is by acquiring more scans, but this was a limited option for ^10^B, with the complete spectrum for one orientation usually consisting of several sub-spectra, each requiring several days of measurement time (see Experimental for further details). Nevertheless, the error ratios of our single-crystal work do not invalidate the claim made by Bray and co-workers [1] about quadrupolar interaction parameters being available from ^10^B spectra to higher precision. This argument was made for polycrystalline samples, where the measurement of the larger span of the inner transitions of spin I=3 (m=0↔±1) was compared to measuring the much smaller broadening of the central transition (CT) of spin I=3/2 (m=+1/2↔−1/2) under second-order effects. However, the situation is fundamentally different for a single crystal, where the positions of the satellite transitions are evaluated to extract the quadrupole parameters. Since these positions cover a much larger frequency range than the comparatively small changes of the CT, the above argument does not apply.

### 3.3. Determination of the Chemical Shift Tensor

To determine the chemical shift tensor, the movement of the centres of gravity (CoGs) of the ±k doublets around the Larmor frequency $\nu_{0}$ are evaluated:

(21) $$\nu_{\mathrm{CoG}}\left(\varphi \right)=\frac{\nu (+k)+\nu (-k)}{2}-\nu_{0}=\nu_{\mathrm{CS}}\left(\varphi \right)+\nu_{\chi }^{\left(2\right)}\left(\varphi \right)$$

**Figure 5:**
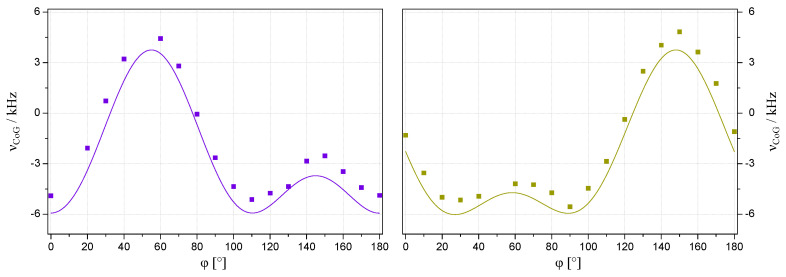
Centres of gravity (CoGs, see Equation (21)) of ^10^B doublets belonging to k=±1/2 transitions, with the lines representing the predicted second-order contributions according to Equation (16).

Here, the individual frequencies ν(±k) are those given by Equation (1). Since the effect of the dipolar interaction as described by $\nu_{\mathrm{DD}}$ leads only to an unspecific broadening of the single-crystal resonance lines, it has been omitted from the above equation; the first-order contributions of the quadrupole interaction with opposing signs ±k cancel each other, leaving only the chemical shift and the second-order contribution. For illustrative purposes, these data points are plotted for two k=±1/2 transitions in Figure 5, together with the second-order contributions calculated from Equation (16) using the χ value of Equation (20).

The application of Equation (16) requires knowledge of the polar coordinates β,α of the magnetic field vector $\vec{b}$ in the principal axes system (PAS) of the Q-tensor. However, for this data fit the movement of $\vec{b}$ is calculated in the crystal frame (CRY), as outlined in Appendix A.2. One possible procedure to derive β,α from the orientation of $\vec{b}$ in the CRY frame is described in Appendix A.4.

**Figure 6:**
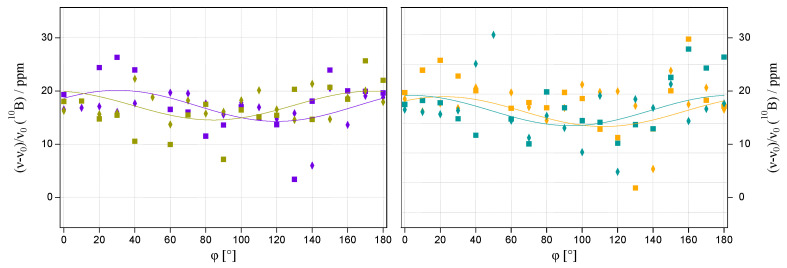
Chemical shift effects on the centres of gravity of the ^10^B doublets for k=±1/2,±3/2 after removing the second-order contributions according to Equation (16). The coding of the transitions by marker shape and of the assigned boron pairs by colour are identical to those used in Figure 4. The drawn lines represent the fit of the chemical shift tensor, with the resulting eigenvalues listed in Table 3.

After subtracting $\nu_{\chi }^{\left(2\right)}$ from the centres of gravity described by Equation (16), the remaining data points are affected by chemical shift only, as plotted for all k=±1/2,±3/2 transitions in Figure 6.

Simultaneous fit of these points according to equations similar to those in Appendix A.2 (but without pre-factor) results in the chemical shift tensor of ^10^B listed in Table 3. Comparison to the previously determined values for ^11^B [20], as listed in the same table, shows that the values are identical for both isotopes within error margins, but that the errors themselves are much larger for ^10^B. As discussed for the quadrupole tensor above, the errors are chiefly caused by the poor signal-to-noise ratio of the individual ^10^B spectra.

### 3.4. Comparison of ^10^B- and ^11^B-NMR Line Widths

**Figure 7:**
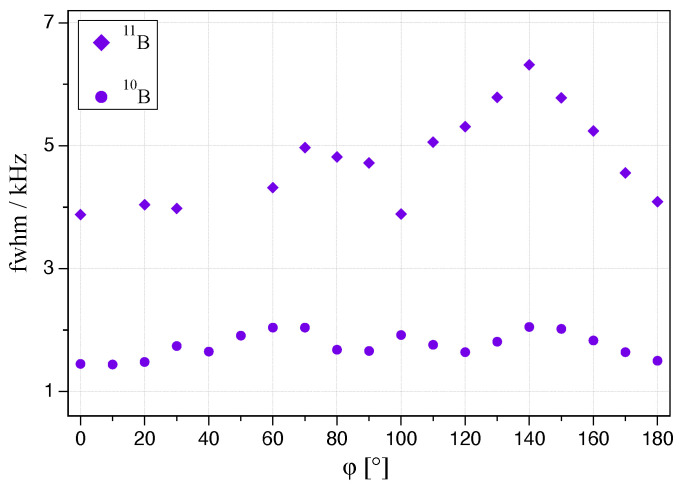
Line widths (full width half maximum-fwhm) of the ^10^B and ^11^B resonances of the B(1,5) pair across their full respective rotation patterns of the hambergite single crystal.

For the comparatively small residual line width of the boron single-crystal resonances, the heteronuclear dipolar interaction as described by Equation (2) has been shown in our previous study to be the dominant cause for broadening of the spectral lines for ^11^B [20]. Resolved dipolar doublets due to direct ^1^H–^11^B couplings could be observed for some crystal orientations, with the doublets disappearing and line widths generally decreasing under application of proton decoupling [20]. Consequently, because of the lower gyromagnetic ratio of ^10^B, those line widths should be even smaller in the ^10^B spectra. Figure 7 shows the full width at half maximum (fwhm) in kHz for both the ^10^B and ^11^B resonances for the B(1,5) pair, acquired for the current paper, across the full respective rotation patterns of the single crystal. It can be seen that some variations of the fwhm show up over the rotation angle because of the orientation dependence of the dipolar interaction. However, averaging the fwhm values over the entire rotation pattern leads to an average ratio of ^10^B/^11^B = 0.410, very close to the theoretical value of 0.335 predicted by Equation (3). Therefore, our single-crystal results fully validate the claim advanced by Bray and co-workers [1] about superior resolution being available from ^10^B spectra as compared to ^11^B whenever the main broadening mechanism is dipolar coupling.

The situation becomes more complex when distributions of chemical shift or quadrupolar interaction are involved. Such distributions are present in glasses [1,2,3,4,5], but may also occur in single crystals of natural minerals, for example as domain distributions caused by crystal mosaicity [36]. The concomitant distribution of the NMR interaction tensor orientation leads to increased NMR line widths, which may in turn show orientation dependence [37]. If these line widths are expressed in kHz, as in Figure 7, then ^10^B again has an advantage over ^11^B for a chemical shift distribution because of the lower Larmor frequency. For the quadrupole interaction, the maximal displacement observable to first order in both single or polycrystalline sample is described by Equation (11). For ^10^B with spin I=3, the largest available value for *k* is $k_{\max}=\pm 5/2$, while for ^11^B with spin I=3/2 it is $k_{\max}=\pm 1$. With the quadrupolar frequency (Equation (6)) of ^11^B being $\nu_{Q}{(}^{11}B)=\chi /2$, for the two isotopes this gives

(22) $$\begin{array}{ccc} \nu_{\chi }^{\left(1,\max\right)}{(}^{10}B)=\pm \frac{1}{4}\chi & \; & \nu_{\chi }^{\left(1,\max\right)}{(}^{11}B)=\pm \frac{1}{2}\chi \end{array}$$

Because χ for ^10^B is about double that of ^11^B, the line broadening caused by a distribution of the quadrupolar interaction is very similar for the two isotopes. In practice, the outer transitions of ^10^B with $k_{\max}=\pm 5/2$ prove difficult to record (we omitted them in our single crystal analysis here), and the signal-to-noise conditions will be even worse for polycrystalline samples; hence, when basing the data analysis on the inner ^10^B transitions with |k|<5/2, the maximal possible displacement (cf. Table 1) is again smaller, and in the presence of distributions should give better spectral resolution compared to ^11^B.

## 4. Conclusions

^10^B-NMR spectroscopy has been applied to a single crystal of the natural mineral hambergite, Be_2_BO_3_OH. In a solid-state NMR spectrum, three doublets are expected for ^10^B with spin I=3, and these were observed for the four inversion-related pairs of boron sites in the crystal structure of hambergite. Evaluation of the full rotation pattern (with the goniometer axis orientation known from a previous study [20]) allowed for determination of both the electrical field gradient (EFG) and the chemical shift tensor. The numerical values are in very good agreement with those derived from ^11^B-NMR spectroscopy of the same sample [20]. The previously suggested notion [1] about inherently better resolution being available from ^10^B in comparison to ^11^B because of the lower gyromagnetic ratio of the former could be confirmed by our single-crystal data.

## 5. Materials and Methods

NMR spectra were recorded on an Avance-III 500 WB spectrometer (Bruker, Karlsruhe, Germany) at LMU Munich, with a Larmor frequency of $\nu_{0}{(}^{10}B)=53.72$ MHz. The sample crystal was glued onto a wooden axis which was spanned into a goniometer mechanics (built by NMR Service GmbH, Erfurt, Germany), permitting its rotation perpendicular to the external magnetic field. This setup was used with a wide-bore static NMR probe (Bruker), with a solenoid coil placed around the sample. Spectra were referenced against the secondary reference of the ^1^H resonance of 1 % Si(CH_3_)_4_ in CDCl_3_. The program Igor Pro [38] was used to extract the NMR interaction tensors from the NMR data, using global fits across several datasets. The obtained tensors in the CRY frame were diagonalised using the online tool Wolfram|Alpha, which is based on the software Mathematica [39]. Eigenvalue errors were estimated according to the method of Nelson [40]. For each crystal orientation, between three and five ^10^B spectra with varying transmitter offset had to be acquired in order to cover the entire spectral range. Using a recycle delay of 600 s and acquiring between 400 (for the stronger inner transitions) and 800 (for weaker outer transitions) scans, recording all resonance frequencies for one particular crystal orientation took between one and three weeks. Acquisition parameters for the ^11^B rotation pattern were identical to those described in [20].

## Figures and Tables

**Table 1:** NMR transition properties for spin I=3 under quadrupolar interaction to the first order (Equation (9)), with the maximal frequencies $\nu_{\chi }^{\left(1,\max\right)}$ defined by Equation (11).

Transition:			Relative
m→m+1	k(m)	$\nu_{\chi }^{\left(1,\max\right)}\left(k\right)$	Intensity
−3→−2	$-\frac{5}{2}$	$-\frac{5}{20}\chi$	3
−2→−1	$-\frac{3}{2}$	$-\frac{3}{20}\chi$	5
−1→0	$-\frac{1}{2}$	$-\frac{1}{20}\chi$	6
0→+1	$+\frac{1}{2}$	$+\frac{1}{20}\chi$	6
+1→+2	$+\frac{3}{2}$	$+\frac{3}{20}\chi$	5
+2→+3	$+\frac{5}{2}$	$+\frac{5}{20}\chi$	3

**Table 2:** Eigenvalues and asymmetry parameter of the electrical field gradient (EFG) tensor V for boron in the crystal structure of hambergite, Be_2_BO_3_OH, as derived from ^10^B- and ^11^B-NMR measurements of a single crystal. The quadrupole moments used for the conversion from the experimentally determined Q tensor are $Q{(}^{10}B)=84.59$ mb and $Q{(}^{11}B)=40.59$ mb [23]. For comparison, the values derived from DFT calculations [20] are also listed.

	^10^B-NMR (This Work)	^11^B-NMR (Ref. [20])	DFT (VASP) (Ref. [20])
$V_{11}$/(V/Å^2^)	−13.05±0.15	−12.970±0.029	−14.792
$V_{22}$/(V/Å^2^)	−13.79±0.30	−14.01±0.08	−15.973
$V_{33}$/(V/Å^2^)	26.84±0.20	26.98±0.05	30.765
$\eta_{V}$	0.028±0.017	0.039±0.005	0.038

**Table 3:** Eigenvalues of the chemical shift tensor δ and the corresponding isotropic chemical shift values for boron in the crystal structure of hambergite, Be_2_BO_3_OH, as derived from ^10^B- and ^11^B-NMR measurements of a single crystal.

	Source	$\delta_{11}$/ppm	$\delta_{22}$/ppm	$\delta_{33}$/ppm	$\delta_{\mathrm{iso}}$/ppm
^10^B	this work	20±4	19±10	12±8	17±8
^11^B	Ref. [20]	19.7±0.9	19.1±0.7	15.4±1.4	18.1±1.0

**Figure A1:**
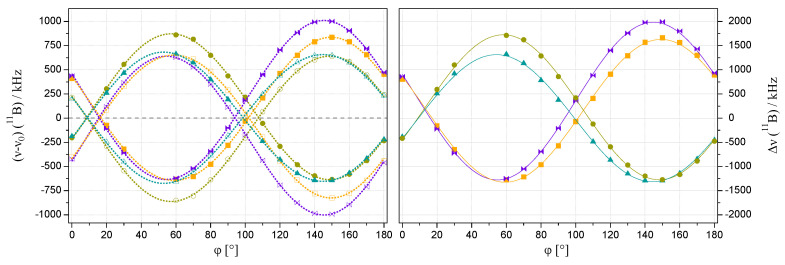
(**Left**): Hambergite rotation pattern of ^11^B (spin I=3/2), additionally recorded to aid the determination of the goniometer axis orientation. The colour coding used for the four magnetically inequivalent boron pairs is identical to that in [20]. (**Right**): Splittings Δν according to Equation (18), with the same colour coding applied.

## Data Availability

The original contributions presented in this study are included in the article. Further inquiries can be directed to the corresponding author.

## Appendix A

### Appendix A.1. Coefficients for Second-Order Quadrupole Interaction

Coefficients for the functions *g* and *f* of Equation (17) (with η being the short notation for $\eta_{Q}$):

$$\begin{array}{cc} A^{\left(2\right)}=-\frac{27}{8}+\frac{9}{4}\eta \cos2\alpha -\frac{3}{8}\eta^{2}\cos^{2}2\alpha \; & \;D^{\left(2\right)}=-\frac{3}{2}+\eta \cos2\alpha -\frac{1}{6}\eta^{2}\cos^{2}2\alpha \\ B^{\left(2\right)}=\frac{30}{8}-\frac{1}{2}\eta^{2}-2\eta \cos2\alpha +\frac{3}{4}\eta^{2}\cos^{2}2\alpha \; & \;E^{\left(2\right)}=\frac{3}{2}-\frac{1}{6}\eta^{2}-\eta \cos2\alpha +\frac{1}{3}\eta^{2}\cos^{2}2\alpha \\ C^{\left(2\right)}=-\frac{3}{8}+\frac{1}{3}\eta^{2}-\frac{1}{4}\eta \cos2\alpha -\frac{3}{8}\eta^{2}\cos^{2}2\alpha \; & \;F^{\left(2\right)}=\frac{1}{6}\eta^{2}-\frac{1}{6}\eta^{2}\cos^{2}2\alpha \end{array}$$

### Appendix A.2. Data Fit Equations and Orientation of the Magnetic Field in the CRY Frame

Data fit equations for the splittings Δν of ^10^B, with the prefactor $F=\frac{1}{10}$ for the transitions with $k=\pm \frac{1}{2}$ and $F=\frac{3}{10}$ for those with $k=\pm \frac{3}{2}$; the subscripts *e*, *c*, *b*, *a* refer to the directions of the $180^{\circ }$ rotation axes connecting the Q-tensors of the four boron sites in hambergite (see Figure 2b), with *e* being the identity operator.

$$\begin{array}{c} \Delta \nu_{e}=F\cdot \left(Q_{xx}\cdot b_{x}^{2}+Q_{yy}\cdot b_{y}^{2}-\left(Q_{xx}+Q_{yy}\right)\cdot b_{z}^{2}+2Q_{xy}\cdot b_{x}b_{y}+2Q_{xz}\cdot b_{x}b_{z}+2Q_{yz}\cdot b_{y}b_{z}\right)\\ \Delta \nu_{c}=F\cdot \left(Q_{xx}\cdot b_{x}^{2}+Q_{yy}\cdot b_{y}^{2}-\left(Q_{xx}+Q_{yy}\right)\cdot b_{z}^{2}+2Q_{xy}\cdot b_{x}b_{y}-2Q_{xz}\cdot b_{x}b_{z}-2Q_{yz}\cdot b_{y}b_{z}\right)\\ \Delta \nu_{b}=F\cdot \left(Q_{xx}\cdot b_{x}^{2}+Q_{yy}\cdot b_{y}^{2}-\left(Q_{xx}+Q_{yy}\right)\cdot b_{z}^{2}-2Q_{xy}\cdot b_{x}b_{y}+2Q_{xz}\cdot b_{x}b_{z}-2Q_{yz}\cdot b_{y}b_{z}\right)\\ \Delta \nu_{a}=F\cdot \left(Q_{xx}\cdot b_{x}^{2}+Q_{yy}\cdot b_{y}^{2}-\left(Q_{xx}+Q_{yy}\right)\cdot b_{z}^{2}-2Q_{xy}\cdot b_{x}b_{y}-2Q_{xz}\cdot b_{x}b_{z}+2Q_{yz}\cdot b_{y}b_{z}\right) \end{array}$$

The magnetic field vector $\vec{b}$ may be written in either Cartesian or spherical coordinates.

(A1) $$\vec{b}=\left(\begin{array}{c} b_{x}\\ b_{y}\\ b_{z} \end{array}\right)=\left(\begin{array}{c} \sin\theta \cos\phi \\ \sin\theta \sin\phi \\ \cos\theta \end{array}\right)$$

In order for the data fit to work, it is obviously necessary to express both $\vec{b}$ and the Q-tensors in the same coordinate system. Usually (including in this work) a frame CRY using the axes of the crystallographic unit cell is chosen. During the experiment, the stepwise rotation of the crystal around the goniometer axis $\vec{g}$ causes a stepwise rotation of $\vec{b}$ in the CRY frame. This movement may be described with the help of two vectors $\vec{u}$ and $\vec{v}$, which are constructed such as to be perpendicular to $\vec{g}$ and to each other. An offset-angle $\varphi_{0}$ is also introduced in order to take the arbitrary zero angle of the experimental data into account:

(A2) $$\vec{b}\left(\varphi_{i}\right)=\vec{v}\sin\left(\varphi_{i}-\varphi_{0}\right)+\vec{u}\cos\left(\varphi_{i}-\varphi_{0}\right)$$

The vectors $\vec{u}$ and $\vec{v}$ are defined with the help of a reference vector, which must be non-parallel to $\vec{g}$. When using the crystallographic *c*-axis as reference, i.e., $\vec{c}=\left(0\;0\;1\right)$, the auxiliary vectors are given by

$$\begin{array}{c} \vec{v}=\frac{1}{\sin\theta_{g}}\left(\vec{g}\times \vec{c}\right)\\ \vec{u}=\vec{v}\times \vec{g}=\frac{1}{\sin\theta_{g}}\left(\vec{g}\times \vec{c}\right)\times \vec{g}=\frac{1}{\sin\theta_{g}}\left(\vec{c}-\vec{g}\cos\theta_{g}\right) \end{array}$$

where $\theta_{g}$ is the polar angle of the goniometer axis $\vec{g}$ in the crystal frame. The above equations make it possible to also use the orientation of $\vec{g}$ as a variable of the data fit. For further details on these fit routines, see [16,20].

### Appendix A.3. ^11^B Rotation Pattern of Hambergite

The ^11^B rotation pattern acquired from the hambergite single crystal used in the current work is shown below in Figure A1; see Section 3.2 for detailed discussion.

### Appendix A.4. Orientation of the Magnetic Field in the Tensor PAS

For every general 3×3 tensor $T_{g}$ with eigenvalues $T_{11}$, $T_{22}$, $T_{33}$, there exists a matrix S such that the following product diagonalises $T_{g}$:

$$S^{-1}\cdot T_{g}\cdot S=S^{-1}\cdot \left(\begin{array}{ccc} T_{xx} & T_{xy} & T_{xz}\\ T_{xy} & T_{yy} & T_{yz}\\ T_{xz} & T_{yz} & T_{zz} \end{array}\right)\cdot S=\left(\begin{array}{ccc} T_{11} & 0 & 0\\ 0 & T_{22} & 0\\ 0 & 0 & T_{33} \end{array}\right)$$

The matrix S is constructed using the eigenvectors $v_{1}$, $v_{2}$, $v_{3}$ associated with the eigenvalues as columns:

$$S=\left(\begin{array}{ccc} v_{1} & v_{2} & v_{3} \end{array}\right)$$

If the tensors $T_{g}$ and S are expressed in the coordinate system where the data evaluation is performed (usually, as in this work, the crystal frame CRY), then the vector of the magnetic field (as given by Equation (A2)) in this CRY frame may be transformed into the principal axes system (PAS) of the tensor $T_{g}$ by the product:

(A3) $${\vec{b}}^{\mathrm{PAS}}\left(\varphi_{i}\right)=S^{-1}\cdot {\vec{b}}^{\mathrm{CRY}}\left(\varphi_{i}\right)$$

Extracting the spherical coordinates of ${\vec{b}}^{\mathrm{PAS}}$ according to Equation (A1) delivers the angles required for the calculation of the quadrupolar contribution to second order (Equation (16)), with α=ϕ and β=θ.

## References

[1] Jellison G.E., Bray P.J. (1976). A determination of the distributions of quadrupole coupling constants in borate glasses using B^10^ NMR. Solid State Comm..

[2] Jellison G.E., Feller S.A., Bray P.J. (1977). NMR powder patterns for integer spin nuclei in the presence of asymmetric quadrupole effects. J. Magn. Reson..

[3] Jellison G.E., Panek L.W., Bray P.J., Rouse G.B. (1977). Determination of structure and bonding in vitreous B_2_O_3_ by means of B^10^, B^11^ and O^17^ NMR. J. Chem. Phys..

[4] Jellison G.E., Bray P.J. (1978). Structural interpretation of B^10^ and B^11^ NMR spectra in sodium borate glasses. J. Non-Cryst. Solids.

[5] Silver A.H., Bray P.J. (1958). Nuclear magnetic resonance absorption in glass. I. Nuclear quadrupole effects in boron oxide, soda-boric oxide, and borosilicate glass. J. Chem. Phys..

[6] Silver A.H., Bray P.J. (1960). NMR study of bonding in some solid boron compounds. J. Chem. Phys..

[7] Bray P.J., Edwards J.O., O’Keefe J.G., Ross V.F., Tatsuzaki I. (1961). Nuclear magnetic resonance studies of B^11^ in crystalline borates. J. Chem. Phys..

[8] Wong Y.-T.A., Bryce D.L. (2018). Recent advances in ^11^B solid-state nuclear magnetic resonance spectroscopy of crystalline solids. Annu. Rep. NMR Spectrosc..

[9] Holland D., Feller S.A., Kemp T.F., Smith M.E., Howes A.P., Winslow D., Kodama M. (2007). Boron-10 NMR: What extra information can it give about borate glasses?. Phys. Chem. Glas. Eur. J. Glass Sci. Technol. B.

[10] Berkowitz J., McConnell M.R., Tholen K., Feller S., Affatigato M., Martin S.W., Holland D., Smith M.E., Kemp T.F. (2009). Elucidation of quadrupole parameters by simulation of ^10^B NMR powder patterns. Phys. Chem. Glas. Eur. J. Glass Sci. Technol. B.

[11] Shadle D., Michalek S., Wilkinson C., Goranson K., Faaborg M., Appler M., Wells J., Bohach G., Affatigato M., Feller S. (2019). A ^10^B NMR study of tetrahedral borons in ring structured borates. Phys. Chem. Glas. Eur. J. Glass Sci. Technol. B.

[12] Penner G.H., Ruscitti B., Reynolds J., Swainson I. (2002). Structure and dynamics of ND_3_BF_3_ in the solid and gas phases: A combined NMR, neutron diffraction and ab initio study. Inorg. Chem..

[13] Smolnikov A.G., Kashnikova M.E., Utkin N.A., Sadykov A.F., Piskunov Y.V., Ogloblichev V.V., Gerashenko A.P., Stashkova L.A., Kazak N.V. (2025). Hyperfine interactions and magnetic order in Co_3_BO_5_ according to ^10,11^B and ^59^Co NMR data. Solid State Nucl. Magn. Reson..

[14] Duong N.T., Kuprov I., Nishiyama Y. (2018). Indirect detection of ^10^B (*I* = 3) overtone NMR at very fast magic angle spinning. J. Magn. Reson..

[15] Vosegaard T. (2021). Single-Crystal NMR spectroscopy. Prog. Nucl. Magn. Reson. Spectrosc..

[16] Bräuniger T. (2024). High-Precision Determination of NMR Interaction Parameters by Measurement of Single Crystals: A Review of Classical and Advanced Methods. Molecules.

[17] Bochkin G.A., Fel’dman E.B., Kuznetsova E.I., Lazarev I.D., Vasil’ev S.G., Volkov V.I. (2020). ^1^H NMR in a quasi-one-dimensional zig-zag spin chain of hambergite, Be_2_BO_3_(OH). J. Magn. Reson..

[18] Vasil’ev S.G. (2021). Investigation of linear and alternating quasi-one-dimensional spin chains in hambergite single crystal by means of 1H NMR. Appl. Magn. Reson..

[19] Bochkin G.A., Fel’dman E.B., Kiryukhin D.P., Kushch P.P., Vasil’ev S.G. (2023). ^1^H multiple quantum NMR in alternating quasi-one-dimensional spin chains of hambergite. J. Magn. Reson..

[20] Steinadler J., Krach G., Witthaut K., Stürzer T., Hochleitner R., Schnick W., Bräuniger T. (2026). Ambiguities in assigning single-crystal NMR data to individual atoms in the crystal structure: A case study of hambergite, Be_2_BO_3_OH, by ^9^Be and ^11^B NMR spectroscopy, XRD measurements and DFT calculations. Magn. Reson. Chem..

[21] Harris R.K., Becker E.D., Cabral de Menezes S.M., Goodfellow R., Granger P. (2002). NMR Nomenclature: Nuclear Spin Properties and Conventions for Chemical Shifts (IUPAC Recommendations 2001). Concepts Magn. Reson..

[22] Cohen M.H., Reif F. (1957). Quadrupole Effects in Nuclear Magnetic Resonance Studies of Solids. Solid State Phys..

[23] Pyykkö P. (2018). Year-2017 nuclear quadrupole moments. Mol. Phys..

[24] Buckingham A.D., Malm S.M. (1971). Asymmetry in the nuclear magnetic shielding tensor. Mol. Phys..

[25] Volkoff G.M., Petch H.E., Smellie D.W.L. (1952). Nuclear electric quadrupole interactions in single crystals. Can. J. Phys..

[26] Zachariasen W.H. (1931). The crystalline structure of hambergite, Be_2_BO_2_(OH). Z. Krist..

[27] Zachariasen W.H., Plettinger H.A., Marezio M. (1963). The structure and birefringence of hambergite, Be_2_BO_2_.OH. Acta Crystallogr..

[28] Burns P.C., Novák M., Hawthorne F.C. (1995). Fluorine-hydroxyl variation in hambergite: A crystal-structure study. Can. Mineral..

[29] Diego Gatta G., McIntyre G.J., Bromiley G., Guastoni A., Nestola F. (2012). A single-crystal neutron diffraction study of hambergite, Be_2_BO_3_(OH,F). Am. Mineral..

[30] Harbison G.S., Kye Y.-S., Penner G.H., Grandin M., Monette M. (2002). ^14^N quadrupolar, ^14^N and ^15^N chemical shift, and ^14^N–^1^H dipolar tensors of sulfamic acid. J. Phys. Chem. B.

[31] Kye Y.-S., Zhao X., Harbison G.S. (2005). Orientation of single crystal using linear approximations to NMR transits. J. Magn. Reson..

[32] Momma K., Izumi F. (2011). VESTA 3 for three-dimensional visualization of crystal, volumetric and morphology data. J. Appl. Crystallogr..

[33] Zeman O.E.O., Hoch C., Hochleitner R., Bräuniger T. (2018). NMR interaction tensors of ^51^V and ^207^Pb in vanadinite, Pb_5_(VO_4_)_3_Cl, determined from DFT calculations and single-crystal NMR measurements, using only one general rotation axis. Solid State Nucl. Magn. Reson..

[34] Zeman O.E.O., Steinadler J., Hochleitner R., Bräuniger T. (2019). Determination of the full ^207^Pb chemical shift tensor of anglesite, PbSO_4_, and correlation of the isotropic shift to lead-oxygen distance in natural minerals. Crystals.

[35] Zeman O.E.O., Bräuniger T. (2022). Quantifying the Quadrupolar Interaction by ^45^Sc-NMR Spectroscopy of Single Crystals. Solid State Nucl. Magn. Reson..

[36] Vinet N., Flemming R.L., Higgins M.D. (2011). Crystal structure, mosaicity, and strain analysis of Hawaiian olivines using in situ X-ray diffraction. Am. Mineral..

[37] Zeman O.E.O., Hochleitner R., Schmahl W.W., Karaghiosoff K., Bräuniger T. (2021). Relationship between ^207^Pb NMR chemical shift and the morphology and crystal structure for the apatites, Pb_5_(AO_4_)_3_Cl, vanadinite (A = V), pyromorphite (A = P) and mimetite (A = As). Am. Mineral..

[38] (2018). Igor Pro 7.

[39] (2024). Mathematica.

[40] Nelson W.H. (1980). Estimation of Errors in Eigenvectors and Eigenvalues from Magnetic Resonance Results by Use of Linear Data-Fitting Techniques. J. Magn. Reson..

